# Rhizospheric soil alter extracellular vesicles in the roots of *Huperzia serrata* and their carried miRNA

**DOI:** 10.3389/fgene.2026.1696647

**Published:** 2026-05-13

**Authors:** Na Li, Li Zhou, Junmin Li

**Affiliations:** 1 School of Pharmacology, Taizhou University, Taizhou, China; 2 Zhejiang Key Laboratory for Restoration of Damaged Coastal Ecosystems, School of Life Sciences, Taizhou University, Taizhou, China; 3 Zhejiang Provincial Key Laboratory of Plant Evolutionary Ecology and Conservation, School of Life Sciences, Taizhou University, Taizhou, China

**Keywords:** extracellular vesicles, Huperzia serrata, MicroRNAs, rhizospheric soil, root

## Abstract

**Introduction:**

As a valuable traditional medicinal plant, it is unclear whether the roots of *Huperzia serrata* produce extracellular vesicles (EVs), or whether the addition of rhizospheric soil influences the characterization of EVs and the microRNAs (miRNAs) involved.

**Methods:**

In this study, we compared EVs from the roots of *H. serrata* grown in rhizospheric soil and sterilized peat moss and sequenced the miRNAs within these EVs.

**Results and discussion:**

EVs from the treated roots of *H. serrata* grown in field rhizospheric soil had a significantly smaller size and higher density compared to those from blank roots grown on sterilized peat moss. The results suggested that the physio-chemical properties or soil microbial communities induced by the addition of rhizospheric soil may play an important role in producing of EVs in the roots of *H. serrata*. Among the 242 known miRNAs, the miR166 family had the most observed miRNAs (27 members), followed by the miR156 (26 members), miR159 (24 members), and miR319 (17 members) families. Principal component analysis revealed a clear separation between the treated and blank samples. A total of 20 differentially expressed miRNAs (DE-miRNAs) were filtered, with 10 up-regulated and 10 downregulated. For 12 DE-miRNAs, 183 target genes were predicted. All miRNAs targeted more than four genes, suggesting that these miRNAs had diverse roles. The predicted target genes included numerous transcription factors and genes with different functions. Furthermore, the function of the predicted target genes was annotated by Gene Ontology (GO) and Kyoto Encyclopedia of Genes and Genomes (KEGG) enrichment analyses. A total of 295 GO terms were enriched, with 157 related to biological processes, 89 to molecular functions, and 49 to cellular components. KEGG analysis showed that the target genes of DE-miRNAs were involved in diverse biological and biochemical processes. Twenty-six target genes were associated with 26 KEGG pathways, and the top five enriched pathways were “Nitrogen metabolism”, “Lipoic acid metabolism”, “Linoleic acid metabolism”, “Propanoate metabolism”, “alpha-Linolenic acid metabolism”. Our results could provide a foundation for the development and application of EVs from the roots of *H. serrata*.

## 1 Introduction

*Huperzia serrata* (Thunb. ex Murray) Trevis. is a perennial medicinal plant belonging to the *Huperzia* genus in the family of Lycopodiaceae. It is a valuable traditional medicinal plant, has garnered extensive attention owing to its abundant alkaloid content, particularly huperzine A (HupA), which is effective for the treatment of Alzheimer’s disease (Lei et al., 2015; Guo et al., 2019). Thus, finding ways to enhance the content of hupA in *H. serrata* is crucial for the conservation and sustainable use of wild *H. serrata* resources.

Extracellular vesicles (EVs), including exosomes, microvesicles, and apoptotic vesicles, are membrane-enclosed vesicular compartments secreted by both prokaryotic and eukaryotic cells (Lotvall et al., 2014). EVs play critical roles in cell-to-cell communication by transporting proteins, lipids, and genetic materials (He et al., 2021). For example, EVs can transfer these molecules to recipient cells through direct fusion with the plasma membrane or via mechanisms such as phagocytosis, micropinocytosis, or endocytosis, activating the stimulator of interferon genes (Kitai et al., 2017). However, most studies on EVs focus on mammalian systems (for example, Kitai et al., 2017), with limited knowledge available regarding plant-derived extracellular vesicles (PDEVs) (Liu et al., 2021; Urzì et al., 2022).

EVs have been isolated from the roots of several plants (De Palma et al., 2020; Urzì et al., 2022), including tomato (Lee et al., 2023), lemon (Raimondi et al., 2019), and grapefruit (Xiao et al., 2018). In addition, previous reports have demonstrated that bacterial and viral infections can affect EV release, and EVs derived from fungi have gained increased attention, emerging as a promising field of research (Liu and Hu, 2023). However, it remains unknown whether *H. serrata* roots produce EVs, and whether the addition of rhizospheric soil can alter the production of EVs in *H. serrata* roots.

MicroRNAs (miRNAs) are a class of small RNAs that mediate the silencing of target genes via base pairing to highly complementary binding sites (Zheng et al., 2020). Recently, several studies have confirmed that EVs can carry miRNAs and regulate gene expression in recipient cells (Cai et al., 2018; Liu et al., 2021). For instance, the gastrointestinal parasite *Heligmosomoides polygyrus* secretes exosomes, a class of EVs, to transport miRNAs into mammalian cells and suppress host immunity (Cai et al., 2018). In this study, we compared the characterization of EVs from *H. serrata* roots grown in rhizospheric soil or sterilized peat moss and sequenced the miRNA within them. Then, the target genes and their functions in *H. serrata* were predicted. We hypothesized that EVs isolated from *H. serrata* roots are likely to be involved in the interaction between *H. serrata* and rhizospheric soil, and may produce specialized miRNAs.

## 2 Materials and methods

### 2.1 Plant materials and treatments

Forty-two *H. serrata* individuals were collected from Kuocang Mountain, Linhai City, Zhejiang Province, China. Whole plants were carefully dug up from the soil, and rhizospheric soil surrounding the roots of *H. serrata* was collected by shaking. All the plants were then transported to the laboratory. Twenty-one stems, approximately 5 cm in length, were cut and propagated in pots (40 cm long×25 cm wide×15 cm high) containing rhizospheric soil and sterilized peat moss at a ratio of 1:1 (v:v) (treated and infected with rhizospheric soil microbes). Twenty-one stems with the similar length were cut and propagated in pots containing sterilized rhizospheric soil and sterilized peat moss at a ratio of 1:1 (v:v) were used as the control (blank and not infected with rhizospheric soil microbes). The pots were placed in a walk-in artificial climate chamber (25 °C, 14 h/20 °C, 10 h; day/night).

### 2.2 Roots collection and EVs isolation

After 95 days, roots of *H. serrata* were harvested and washed with sterilized distilled water. Seven *H. serrata* roots were combined to form one sample, and a further six samples were prepared for the blank and treated experiments. Every root sample was divided into two parts. One part was used for EV isolation and the other for transcriptomic analysis.

The EVs were extracted according to the previous methods and modified (Teng et al., 2018; Shu et al., 2024; 2025). In brief, the roots were homogenized with a buffer solution (20 mM MES hydrate, 2 mM CaCl_2_, 0.1 M NaCl, pH 6.0) for 5 min and then centrifuged at 10,000 g for 60 min to collect the apoplastic fluid. The cellular debris in the apoplastic fluid was removed by centrifugation at 10,000 g for 60 min, followed by filtration through a 0.22 μm filter. Next, the supernatants were transferred to new ultracentrifuge tubes and centrifuged at 120,000 g for 1 h to obtain the pellet. The pellet was resuspended in 200 μL DEPC-treated PBS buffer for further analysis. The purity of the six EV samples was indicated by the A_260_/A_280_ ratio, as measured by BioPhotometer Plus 6,132 (Eppendorf AG, Hamburg, Germany). The size and concentration of the six EVs samples were measured using NanoSight NS300 Nanoparticle Tracking Analysis (Malvern Panalytical Ltd., Worcestershire, UK). To indicate the stability of EV particle size, the size distribution width coefficient was calculated as (D90-D10)/D50.

### 2.3 Electron microscopy detection of EVs

EVs were detected using transmission electron microscopy. In brief, a 20 μL suspension was dropped onto a copper mesh and allowed to adsorb naturally for 5–10 min. Excess liquid was removed using filter paper strips. After slightly drying, 20 μL of 2% phosphotungstic acid solution was added to the samples on the copper mesh and incubated for 3–5 min. Excess liquid droplets were removed with filter paper strips. The samples were air-dried under incandescent lamps and then observed under a transmission electron microscope (HT7700, Hitachi Ltd., Tokyo, Japan). For every sample, five visual fields were observed and photographed.

### 2.4 Small RNA sequencing and data analysis

Total RNA of the six EVs samples was extracted from the roots of *H. serrata* using the RNAiso Plus kit (TaKaRa, China) according to the manufacturer’s instructions. The TruSeq Small RNA Sample Prep Kit (Illumina, California, USA) was used for the miRNA library construction. The enriched library was then amplified by polymerase chain reaction, with sequencing adapters and index sequences added. The library was purified by 1.2% gel electrophoresis, yielding clear bands. After that, the Agilent High Sensitivity DNA Kit with an Agilent 2100 Bioanalyzer (Agilent Technologies, California, USA) was used to assess the library, which was expected to show only a single peak, with no adapters. Finally, the library was qualified using the Quant-iT PicoGreen dsDNA Assay Kit (Thermo Fisher Scientific, Massachusetts, USA), and the samples were sequenced on the Illumina platform. The miRNA sequencing was performed by Yanzai Biotechnology (Shanghai, China) as previously reported (Chen et al., 2021). The raw data have been deposited in Genbank under the accession number PRJNA1228355.

After removing adapter contaminants and low-quality tags from the raw data, clean reads ranging from 18 to 30 nt were selected for analysis. These clean reads were aligned to the *Selaginella moellendorffii* genome by Bowtie (Langmead et al., 2009). The mapped small RNA sequences were then searched against the miRBase 22.0 database to identify known miRNAs. The expression levels of miRNAs were normalized based on transcripts per million (TPM) values (Zhou et al., 2010).

The putative target genes of the DE-miRNAs were predicted by psRobot_tar in psRobot (http://plantgrn.noble. org/psRNATarget/?function = 3). Then, the functions of the predicted target genes were annotated through Gene Ontology (GO) analysis using the Goseq R package (Young et al., 2010) and Kyoto Encyclopedia of Genes and Genomes (KEGG) analysis (Kanehisa et al., 2008). KOBAS software was used to test the statistical enrichment of the target genes, with a *p*-value <0.05 considered as significantly enriched (Mao et al., 2005).

### 2.5 Real-time quantitative PCR (RT-qPCR)

To corroborate sRNA-seq data, five representative DE-miRNAs (hse-miR850, hse-miR166c-3p, hse-miR166-3p, hse-miR482e-5p, hse-miR3630-3p) and their top predicted target genes were selected for RT-qPCR verification. RT-qPCR was performed on ABI ViiA™7 Real-Time PCR System (Life Technology Inc., New York, USA) by using PimeScript™RT Master Mix according to the manufacturer’s protocols (Takara Bio Inc., Dalian, China). The specific primers for miRNA and target genes were designed using Primer Premier 5.0 (Supplementary Table S1). A constitutively expressed gene *18S rRNA* and miR39 was used as references control for the target gene and miRNA, respectively (Perge et al., 2017). The relative expression values of the genes were calculated using formula 2^−ΔΔCt^. For all RT-qPCRs, three biological replicates and three technical replicates were utilized.

### 2.6 Statistical analysis

To test whether the characteristics of EVs changed after the addition of rhizospheric soil, the size and density of EVs in the treatment and control groups were evaluated using a t-test to determine whether there were any significant differences. Statistical analyses were performed using SPSS Statistics 20 (IBM Corp., Armonk, NY, USA). Differentially expressed miRNAs (DE-miRNAs) between the treated and blank libraries were analyzed using the DESeq R package (Love et al., 2014). The DE-miRNAs were determined under the thresholds of |log_2_ (fold change)| ≥ 1 and *p*-value *<* 0.05.

## 3 Results

### 3.1 Phenotypic characteristics of EVs

**FIGURE 1:**
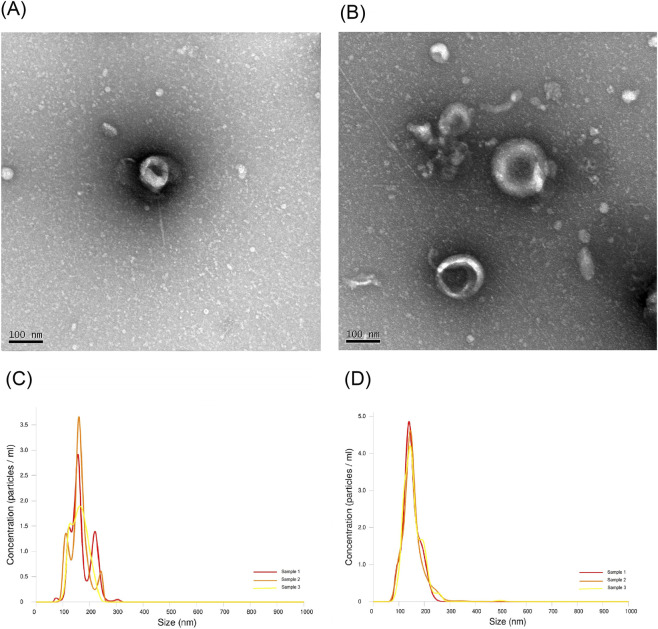
Transmission electric micrographs of EVs from blank **(A)** and treated **(B)** roots of *H. serrata* with the magnification of ×100,000, respectively. Size and concentration of three EVs samples from blank **(C)** and treated **(D)** roots of *H. serrata*. “Blank” refers to *H. serrata* grown in sterilized rhizospheric soil and sterilized peat moss; “Treated” refers to *H. serrata* grown in rhizospheric soil and sterilized peat moss.

All EVs isolated from the blank and treated roots of *H. serrata* showed typical cup-shaped morphology (Figures 1A,B). The high purity was indicated by an A_260_/A_280_ ratio of 1.97 versus 1.93. The size of EVs from the treated roots of *H. serrata* was significantly smaller compared to those from the blank roots (153.03 ± 2.84 nm vs. 164.00 ± 3.61 nm, t = 4.78, p = 0.003) (Figures 1C,D). The size distribution width coefficient of EVs from the blank and treated roots of *H. serrata* were 0.577 and 0.617, respectively, indicating a narrow particle size distribution and good particle uniformity. In addition, the concentration of EVs was significantly higher in the treated roots of *H. serrata* than in the blank roots (7.506 ± 0.098 ×10^7^ particles/μg protein vs. 6.672 ± 0.402 ×10^7^ particles/μg protein, t = 4.456, p = 0.004).

### 3.2 Overview of small RNA sequencing

A total of 184.3 megabytes of raw reads were obtained from the six libraries. After removing low-quality reads and adapters, 145.6 megabytes of clean reads remained, with the following distribution: 24,299,099, 24,561,454, 24,783,370, 23,919,469, 23,824,499, and 24,221,356 clean reads in the blank 1, blank 2, blank 3, treated 1, treated 2 and treated 3 libraries, respectively (Table 1). The length of the clean reads predominantly ranged from 15 to 30 nt (Supplementary Figure S1). On average, 33.81% of the clean reads were mapped to the genome of *Selaginella moellendorffii* (Supplementary Table S2). Approximately 1% of the clean reads aligned to known miRNA, with 1439, 1953, 1456, 1557, 1755, and 1738 miRNA reads in the blank 1, blank 2, blank 3, treated 1, treated 2, and treated 3 libraries, respectively.

**TABLE 1:** Summary of small RNA sequencing and annotation in this study.

Sequencing metrics	Blank 1	Blank 2	Blank 3	Treated 1	Treated 2	Treated 3
Raw reads (M)	28.82	28.96	27.72	32.62	33.15	33.12
Clean reads (M)	24.30	24.56	24.78	23.92	23.82	24.22
Cis-reg (M)	14.13 (0.58%)	9.24 (0.38%)	6.76 (0.27%)	1.73 (0.72%)	1.97 (0.82%)	2.01 (0.83%)
Others (M)	18.63 (0.77%)	16.66 (0.68%)	11.83 (0.48%)	32.06 (1.34%)	29.26 (1.23%)	29.84 (1.23%)
rRNA (M)	12.15 (0.5%)	10.30 (0.42%)	5.09 (0.21%)	19.96 (0.83%)	19.38 (0.81%)	16.92 (0.7%)
snRNA (M)	7.74 (0.32%)	6.19 (0.25%)	4.83 (0.19%)	15.20 (0.64%)	18.55 (0.78%)	13.88 (0.57%)
tRNA (M)	1.35 (0.06%)	1.17 (0.05%)	0.89 (0.04%)	3.41 (0.14%)	4.24 (0.18%)	2.99 (0.12%)
Gene	100	75	135	4584	3173	3722
Repeat (M)	716.10	750.32	776.94	541.59	561.09	571.95
miRNA	1439	1953	1456	1557	1755	1738
Unannotated reads (M)	1659.67	1662.05	1671.85	1761.94	1729.78	1765.92

“Blank” refers to EVs, from the roots of *H. serrata* grown in sterilized rhizospheric soil and sterilized peat moss; “Treated” refers to EVs, from the roots of *H. serrata* grown in rhizospheric soil and sterilized peat moss.

### 3.3 Identification of known miRNAs

**FIGURE 2:**
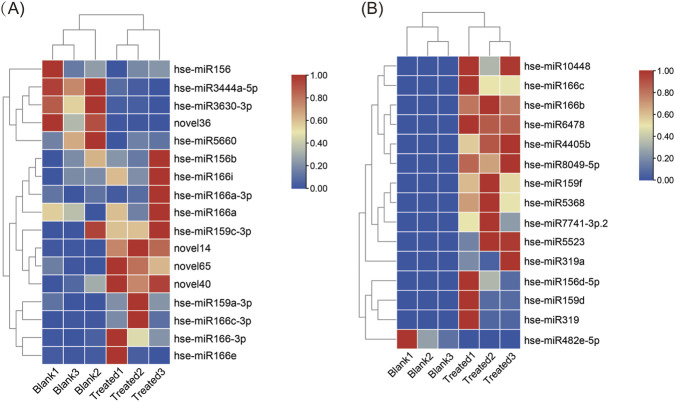
Common and sample-specific miRNAs identified in the six libraries. **(A)** 17 common miRNAs identified in all six libraries. **(B)** miRNAs specific to blank or treated samples. “Blank” refers to EVs from the roots of *H. serrata* grown in sterilized rhizospheric soil and sterilized peat moss; “Treated” refers to EVs from the roots of *H. serrata* grown in rhizospheric soil and sterilized peat moss.

Using a BLAST search against the miRNA database, miRbase (version 22.0), a total of 242 known miRNAs were identified in the six libraries (Supplementary Table S3). Nucleotide bias analysis of known miRNAs revealed a high frequency of uracil at the 5′-end (Supplementary Figure S2). Most of the known miRNAs (132 out of 242) were 21 nt in length, followed by 22 nt (35 miRNAs) and 20 nt (28 miRNAs). The miR166 family contained the most abundant miRNAs, with 27 members, followed by the miR156 (26 members), miR159 (24 members), and miR319 (17 members) families. Among the known miRNAs, 17 were detected in all six libraries, while 15 were specific in the blank or treated samples (Figure 2; Supplementary Table S4). Specifically, one miRNA (hse-miR482e-5p) was specific to blank samples, whereas 14 known miRNAs were specific to treated samples, including hse-miR10448, hse-miR156d-5p, hse-miR4405b, hse-miR5368, hse-miR5523, hse-miR6478, hse-miR7741-3p.2, hse-8049-5p, hse-miR159f, hse-miR159d, hse-miR166b, hse-miR166c, hse-miR319, and hse-miR319a (Figure 2B; Supplementary Table S4).

### 3.4 Analysis of DE-miRNAs

**FIGURE 3:**
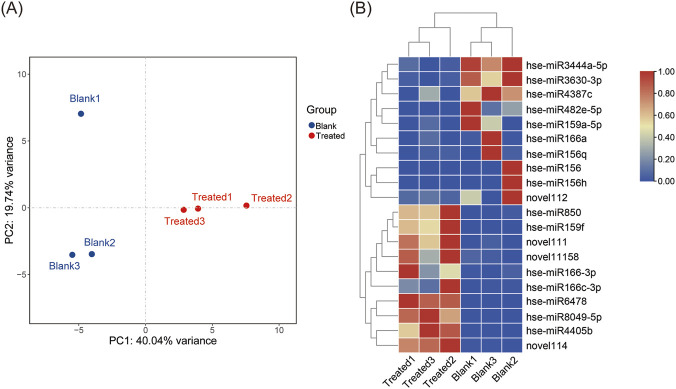
PCA analysis of the identified miRNAs **(A)** and the heatmap of DE-miRNAs **(B)**. “Blank” refers to EVs from the roots of *H. serrata* grown in sterilized rhizospheric soil and sterilized peat moss; “Treated” refers to EVs from the roots of *H. serrata* grown in rhizospheric soil and sterilized peat moss.

To identify the DE-miRNAs involved in pathogen infection, the miRNA profiles were analyzed between treated (Treated 1, 2, 3) and blank (Blank1, 2, 3) samples. Principal component analysis revealed a clear distinction between the treated and blank samples (Figure 3A). A total of 20 DE-miRNAs were filtered based on the thresholds of |log_2_FC| > 1 and *p*-value <0.05, including 10 up-regulated and 10 downregulated miRNAs (Figure 3B; Supplementary Table S5).

### 3.5 Prediction and functional analysis of miRNA-targeted genes

To elucidate the function of DE-miRNAs, their target genes were predicted by psRobot. A total of 431 target genes were predicted for 14 DE-miRNAs (Supplementary Table S6). Each miRNA targeted more than four genes, indicating their diverse regulatory roles. The predicted target genes included numerous transcription factors and genes with distinct functions. For example, the SQUAMOSA Promoter-Binding Protein-Like (SPL) transcription factor family proteins (SPL2/3/4/5/9/10/11/13A/13B/15) were predicted to be modulated by hse-miR156h and hse-miR156 (Supplementary Table S3). The F-box and leucine-rich repeat domain-containing proteins were also targeted by hse-miR156h. MYB domain proteins (MYB101/104/120/33/65/81) were modulated by hse-miR159a and hse-miR159f. WRKY38 was predicted to be targeted by hse-miR3444a-5p. Other predicted targets included calcium-dependent phospholipid-binding copine family protein, DHHC-type zinc finger family protein, and transmembrane protein (putative, DUF677), which were targeted by hse-miR5368. Additionally, peptidase M20/M25/M40 family proteins were targeted by hse-miR482e-5p (Supplementary Table S6).

**FIGURE 4:**
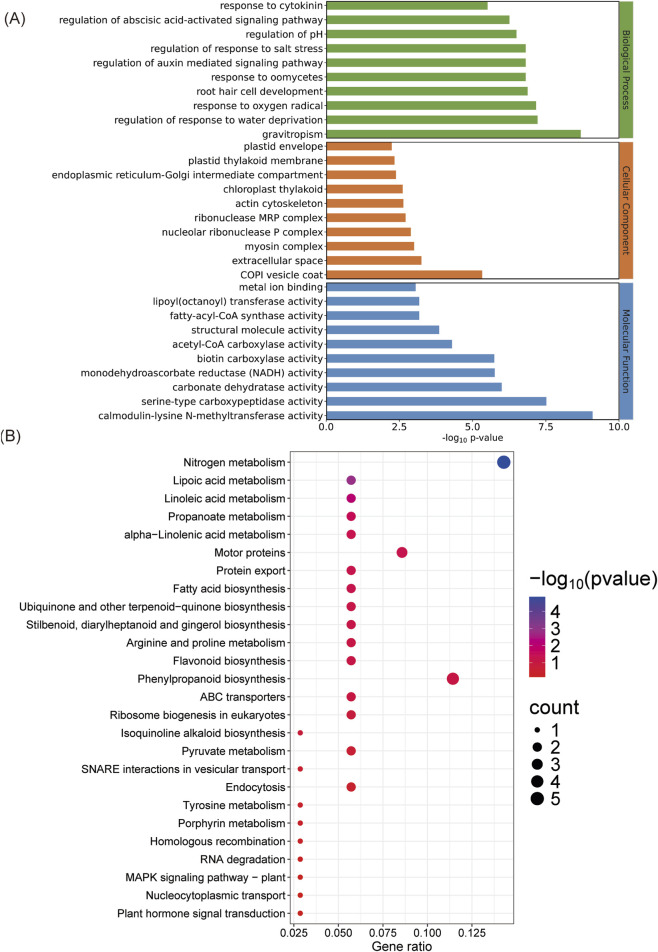
Functional enrichment analysis of the predicted target genes. **(A)** GO enrichment; **(B)** KEGG enrichment. The gene ratio is defined as the number of enriched genes divided by the total number of genes annotated to the corresponding KEGG pathway. The size of the dots indicates the number of DEGs between the treated and blank groups, and the color of the dots reflects the p-value.

Furthermore, the function of the predicted target genes was annotated by GO and KEGG enrichment analysis. A total of 295 GO terms were enriched, including 157 classified under the biological process category, 89 under the molecular function category, and 49 under the cellular component category (Supplementary Table S7). Within the biological process category, most target genes were involved in the gravitropism (9 genes), regulation of response to water deprivation (6 genes), root hair cell development (6 genes), response to oomycetes (6 genes), regulation of auxin mediated signaling pathway (6 genes), regulation of response to salt stress (6 genes), regulation of abscisic acid-activated signaling pathway (6 genes). In the molecular function category, metal ion binding (30 genes) was the most enriched GO term. In the cellular component category, most genes were enriched in extracellular space (6 genes) and chloroplast thylakoid (6 genes) (Figure 4A).

KEGG analysis revealed that the target genes of DE-miRNAs were involved in diverse biological and biochemical processes. Twenty-six target genes were associated with 26 KEGG pathways, 7 of which were significantly enriched (*p*-value <0.05, Supplementary Table S8). These included “Nitrogen metabolism”, “Lipoic acid metabolism”, “Linoleic acid metabolism”, “Propanoate metabolism”, “alpha-Linolenic acid metabolism”, “Motor proteins”, and “Protein export” (Figure 4B).

### 3.6 RT-qPCR verification

**FIGURE 5:**
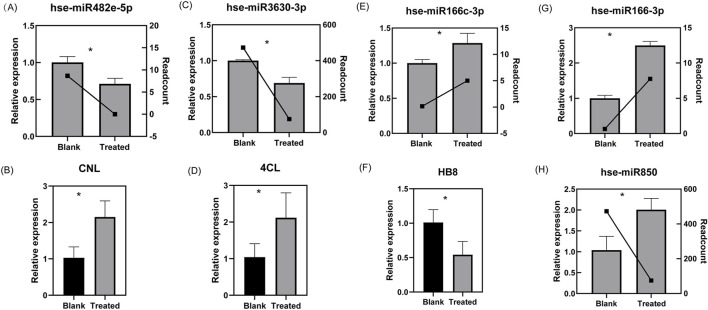
RT-qPCR validated the differentially expressed miRNAs and their corresponding predicted target genes in the EVs from the roots of *H. serrata* infected or uninfected with rhizospheric soil microbes. **(A)** hse-miR482-5p; **(B)** the target gene CNL of hse-miR482e-5p; **(C)** hse-miR3630-3p; **(D)** the target gene 4CL of hse-miR3630-3p; **(E)** hse-miR166c-3p; **(F)** hse-miR166-3p; **(G)** the target gene HB8 of hse-miR166c-3p and hse-miR166-3p; **(H)** hse-miR850. The bar graphs show the relative expression as measured by RT-qPCR, and the line charts are the expression estimated obtained by RNA-seq. “Blank” refers to EVs from the roots of *H. serrata* grown in sterilized peat moss; “Treated” refers to EVs from the roots of *H. serrata* grown in rhizospheric soil. *p < 0.05, independent-samples t-test.

The results of RT-qPCR demonstrated that hse-miR850, hse-miR166c-3p, and hse-miR166-3p expression levels were significantly upregulated in the EVs from the treated roots, whereas hse-miR482e-5p and hse-miR3630-3p were significantly downregulated. These expression patterns are consistent with the trends observed in the sRNA-seq data. Subsequently, quantitative analysis of the predicted target genes revealed that the transcript levels of *HB8*, which was targeted by both hse-miR166-3p and hse-miR166c-3p, was significantly reduced in the Treat group. In contrast, the expression of *CNL* (a target of hse-miR482e-5p) and *4CL* (a target of hse-miR3630-3p) was significantly increased. The inverse correlation between the expression of these miRNAs and their respective target genes not only supports the predicted regulatory relationships but also validates the reliability of the sRNA-seq dataset (Figure 5).

## 4 Discussion

Recent studies have shown that EVs can be released by plants (Urzì et al., 2022; De Palma et al., 2020; Raimondi et al., 2019; Xiao et al., 2018). In this study, we found that EVs were present in the root of *H. serrata* regardless of whether they were grown in field rhizospheric soil or sterilized peat moss. It has been documented that EVs might play important roles as mediators in plant–microbe interactions (Rutter and Innes, 2017; Liu et al., 2021). For example, Rutter and Innes (2017) have found that pathogen infection increases PDEV secretion. In this study, we found that EVs isolated in the roots of *H. serrata* grown in field rhizospheric soil were significantly smaller in size and higher in density than those in roots grown on sterilized peat moss. This suggests that the physio-chemical properties or soil microbial communities induced by the addition of rhizospheric soil could influence the release and properties of EVs from the roots of *H. serrata*, as well as the miRNAs they carry. Our results could provide a foundation for the development and application of EVs from the roots of *H. serrata*.

PDEVs carry miRNA, which can move from cell to cell and induce various effects (Cai et al., 2018). In plants and animals, miRNAs regulate the expression of protein-coding genes by affecting mRNA translation or stability (Rhoades et al., 2002), with nearly half of the target genes being transcription factors (Mallory and Vaucheret, 2006). In this study, a total of 242 known miRNAs, including miR166, miR156, miR159, and miR319, were identified across all six EV samples extracted from the roots of *H. serrata*. Given the known function of miRNAs, these miRNAs carried by EVs might play important roles in various developmental processes and stresses responses. For example, miR166s have been found to be involved in the modulation of various developmental processes via negatively mediating their target genes in plants, including shoot apical meristem development, organ polarity, seed development, vascular patterning of shoot, root development, and nutrient ion uptake, and response to abiotic and biotic stresses (Li et al., 2017); and miR156 regulates grain yield by modulating shoot architecture, seed germination, and grain size (Jiao, 2010; Miao et al., 2019); miR159 regulates plant growth and development, as well as pathogen defense responses (Allen et al., 2007; Zheng et al., 2020). In this study, hse-miR166-3p and hse-miR166c-3p were upregulated, while hse-miR166a were downregulated, suggesting their distinct roles in the response of plants to pathogens; hse-miR156h, hse-miR156q and hse-miR156 were downregulated and predicted to target *SQUAMOSA Promoter-Binding Protein-Like* (*SPL*) gene *2/3/4/5/9/10/11/13A/13B/15,* suggesting their important roles in another development and the regulation of the timing of transition from vegetative to reproductive phase; hse-miR156h targeted *PDE334* and *PSB27* genes, and KEGG enrichment analysis showed that these two target genes were enriched in “photosynthesis”, suggesting that the possible involvement of EVs in plants growth; hse-miR159a-5p was downregulated, while hse-miR159f was upregulated and predicted to target MYB domain proteins (MYB101/104/120/33/65/81), suggesting their important roles in the pathogen defense response. Furthermore, GO enrichment analysis of the target genes of the rhizospheric soil specific miRNAs also showed that most of the target genes were related to root development such as root hair cell development and regulation of auxin activated signaling pathway, and stresses responses such as regulation of response to salt stress.

We also found that one miRNA (hse-miR482e-5p) was specific to EVs from the blank roots, while 14 known miRNAs were specific to EVs from the treated roots, such as hse-miR10448, hse-miR156d-5p, hse-miR4405b, hse-miR5368, hse-miR5523, hse-miR6478, hse-miR7741-3p.2, hse-8049-5p, hse-miR159f, hse-miR159d, hse-miR166b, hse-miR166c, hse-miR319, and hse-miR319a. These results suggest that the specific miRNAs may contribute to the interactions between rhizospheric soil microbes and the roots of *H. serrata*. Moreover, PDEVs are also known to target mammalian cells, exhibiting anti-inflammatory and anti-cancer properties (Urzì et al., 2022). For example, EVs derived from ginseng have been reported to influence inflammatory bowel disease and colitis-associated cancer (Cao et al., 2019). In addition, EVs derived from *Moringa oleifera* seeds, which carry plant miRNAs, can naturally penetrate human tumor cells and exhibit pro-apoptotic effects (Potestà et al., 2020). Further studies are needed to investigate the potential role of miRNAs in the growth of *H. serrata* roots, and to clarify their functions.

It has been verified that the symbiotic relationship between *H. serrata* and its endophytic fungi can influence the growth of *H. serrata* and the synthesis of its metabolites (Cao et al., 2021; Shen et al., 2022). The fungus *Shiraia* sp. Slf14 was isolated from *H. serrata* (Zhu et al., 2010) and was found to produce HupA (Li et al., 2007; Ju et al., 2009). In this study, although we did not find the relationship between the microbial infection and HupA production, KEGG enrichment analysis of the target genes of the rhizospheric soil specific miRNAs showed that most of the target genes were related to the biosynthesis of metabolites, such as those involved in nitrogen metabolism, lipoic acid metabolism, linoleic acid metabolism, propanoate metabolism, alpha-Linolenic acid metabolism. Seo et al. (2023) found that ginseng-derived EVs contained elevated proportions of Rb1 and Rg1 ginsenosides, demonstrating greater efficacy in inhibiting osteoclast differentiation. As an innovative form of herbal medicine, PDEVs exhibit their therapeutic potential due to their unique phospholipid bilayer structure, which encapsulates bioactive molecules including proteins, lipids, RNAs, and metabolites (Han et al., 2025). Further studies are needed to measure the metabolites in EVs isolated from *H. serrata* to ascertain whether they carry medicinal chemical compounds and have a medicinal effect.

Shu et al. (2025) suggested that plant EVs efficiently transporting miRNAs to microorganisms via the temporary immersion bioreactor system. If EVs from *H. serrata* roots can regulate the metabolism of rhizosphere microorganisms by releasing miRNAs (e.g., promoting beneficial bacteria to synthesize growth hormones), which in turn feeds back to the production of plant EVs, forming an interaction cycle. Further studies are needed to investigate the role of microbial taxa in producing EVs in the roots of *H. serrata*, as well as their correlation with metabolite biosynthesis.

## 5 Conclusion

In this study, we found that the addition of soil rhizospheric soil could alter the size and density of the EVs in the roots of *H. serrata,* suggesting that the physio-chemical traits or soil microbial communities induced by the addition of rhizospheric soil could play a certain role in EVs’ production. A total of 242 known miRNAs, including hse-miR166, hse-miR156, hse-miR159, and hse-miR319, were identified in *H. serrata* EVs. Fourteen known miRNAs were found to be specific to EVs extracted from the roots of *H. serrata* grown in the rhizospheric soil and sterilized peat moss. GO and KEGG enrichment analysis revealed that most of the target genes were associated with root development, stresses responses and metabolites biosynthesis. Further research is required to determine the specific role of soil microbial taxa in the rhizospheric soil in the production of EVs by using amplicon sequencing technology, as well as the direct effect of EVs on microorganisms by using co-culture technology.

## Data Availability

The datasets presented in this study are publicly available. This data can be found at the NCBI repository with the accession number PRJNA1228355: https://www.ncbi.nlm.nih.gov/bioproject/PRJNA1228355/.

## Supplementary material

SUPPLEMENTARY FIGURE S1 Length distribution of sRNAs in the six libraries.

SUPPLEMENTARY FIGURE S2 The base distribution of sRNAs in the six libraries.

SUPPLEMENTARY FIGURE S3 Melting curves of primers.
