# Supplementary figures and images for: Rhizospheric soil alter extracellular vesicles in the roots of Huperzia serrata and their carried miRNA
Source: Front Genet. 2026 May 13;17:1696647. doi: 10.3389/fgene.2026.1696647 (PMC13211858; doi:10.3389/fgene.2026.1696647)

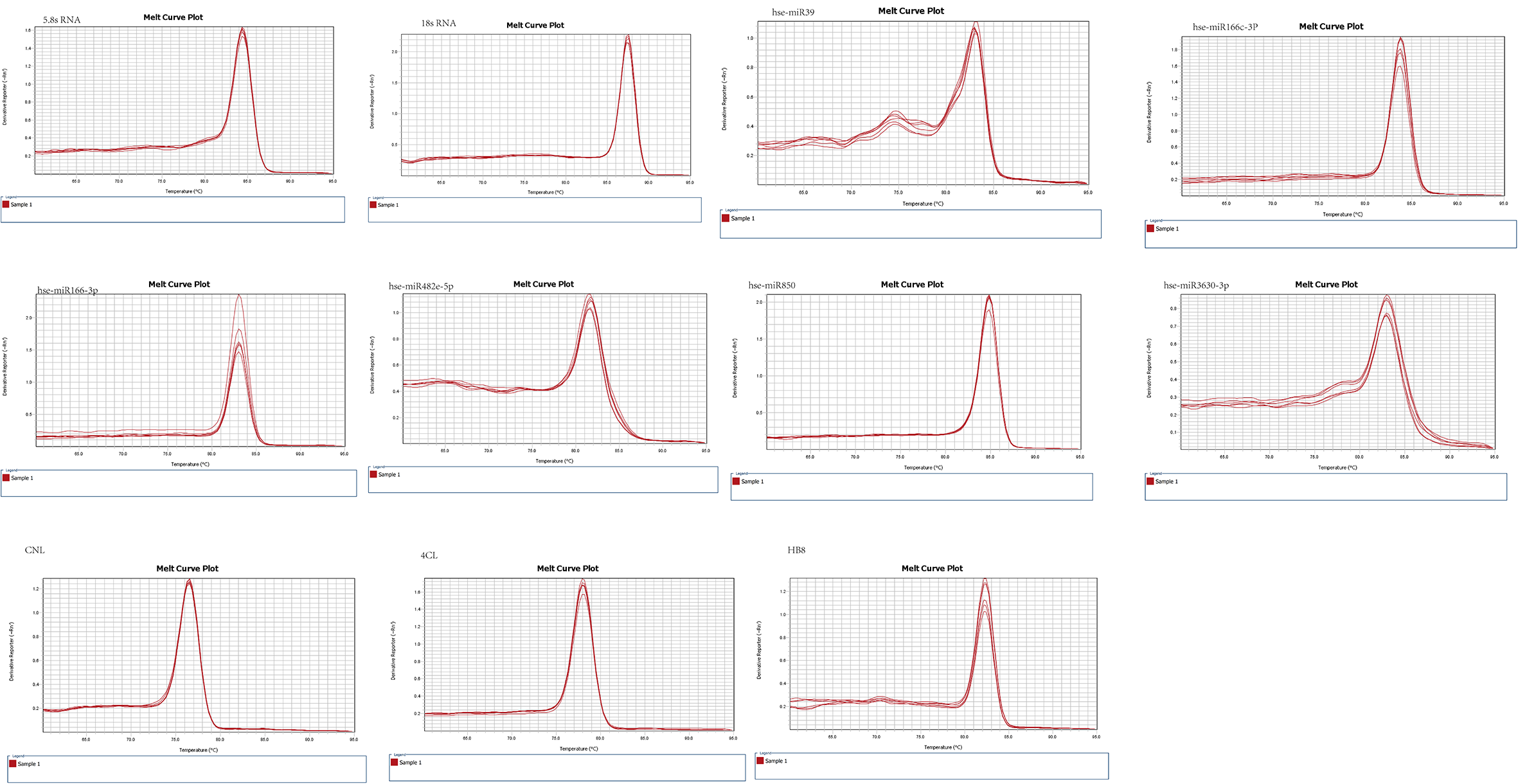

Supplement: Supplementary file 3 [file Image3.tif]

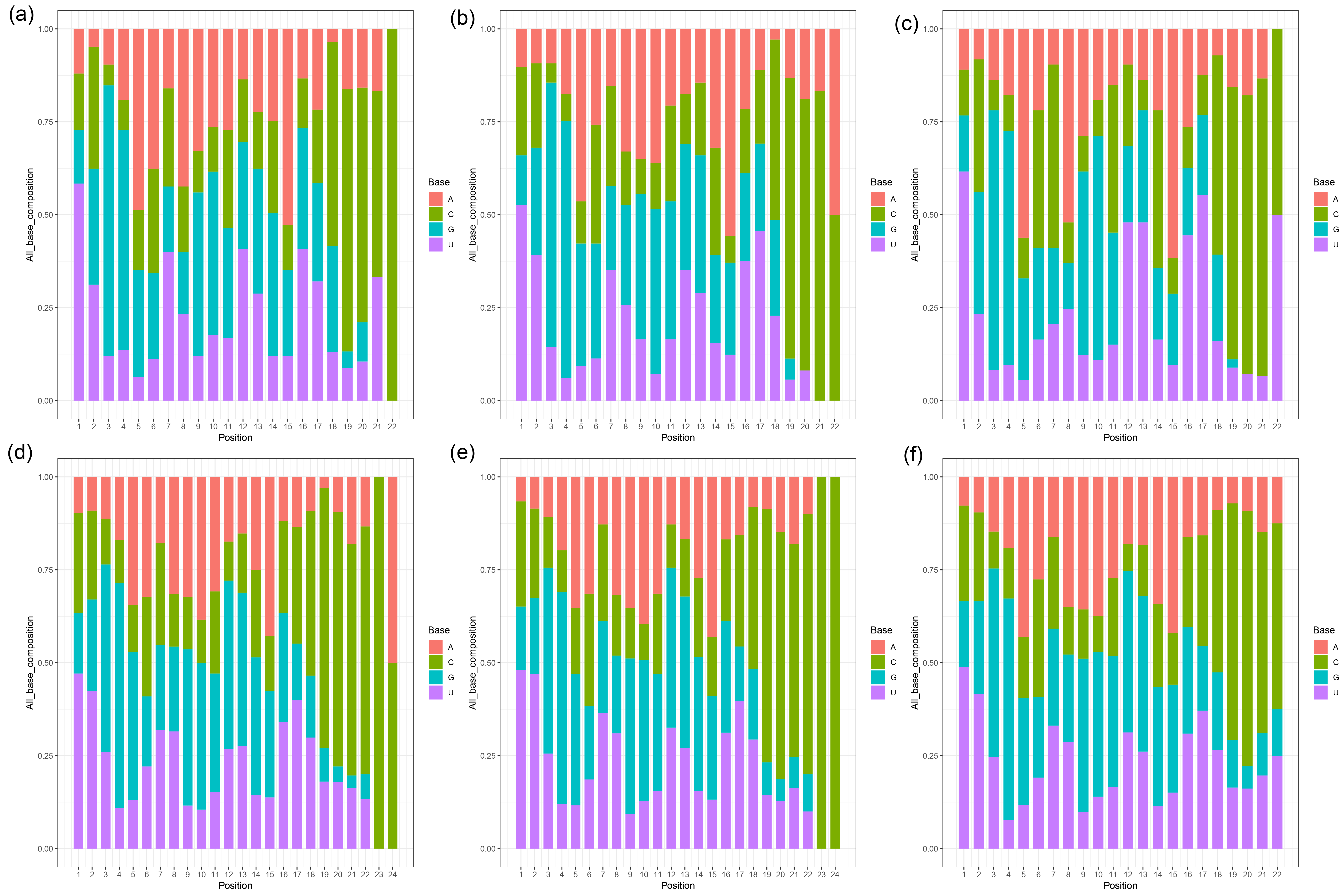

Supplement: Supplementary file 4 [file Image2.tif]
